# Experimental investigation on utilization of *Sesbania grandiflora* residues through thermochemical conversion process for the production of value added chemicals and biofuels

**DOI:** 10.1038/s41598-024-57040-y

**Published:** 2024-03-27

**Authors:** Kedri Janardhana, C. Sowmya Dhanalakshmi, K. T. Thilagham, Santhosh Kumar Chinnaiyan, H. P. Jai Shanker Pillai, T. Sathish, Ümit Ağbulut, Kumaran Palani, Melvin Victor De Poures

**Affiliations:** 1 Department of Electrical Engineering, Faculty of Engineering, Dayalbagh Educational Institute (Deemed to be University), Dayalbagh, Agra, Uttar Pradesh 282005 India; 2 Department of Mechanical Engineering, SNS College of Technology, Coimbatore, Tamil Nadu 641035 India; 3 Department of Metallurgical Engineering, Government College of Engineering, Salem, Tamil Nadu 636011 India; 4 Faculty of Pharmacy, Karpagam Academy of Higher Education, Eachaanari, Coimbatore, Tamil Nadu 641021 India; 5 Department of Microbiology, Assam Downtown University, Panikahiti, Guwahati, Assam 781026 India; 6 Department of Mechanical Engineering, Saveetha School of Engineering, SIMATS, Chennai, Tamil Nadu India; 7 Mechanical Engineering, Faculty of Mechanical Engineering, Yildiz Technical University, 34349 Istanbul, Türkiye; 8 Department of Mechanical Engineering, Wolaito Sodo University, P. O. Box No. 138, Soddo, Ethiopia

**Keywords:** Agricultural residues, Fast pyrolysis, Fluidized bed, Biofuel, Characterization, Biodiesel, Mechanical engineering

## Abstract

All the countries in the world are now searching for renewable, environmentally friendly alternative fuels due to the shortage and environmental problems related with the usage of conventional fuels. The cultivation of cereal and noncereal crops through agricultural activities produces waste biomasses, which are being evaluated as renewable and viable fossil fuel substitutes. The thermochemical properties and thermal degradation behavior of *Sesbania grandiflora* residues were investigated for this work. A fluidized bed reactor was used for fast pyrolysis in order to produce pyrolysis oil, char and gas. Investigations were done to analyze the effect of operating parameters such as temperature (350–550 °C), particle size (0.5–2.0 mm), sweeping gas flow rate (1.5–2.25 m^3^/h). The maximum of pyrolysis oil (44.7 wt%), was obtained at 425 °C for 1.5 mm particle size at the sweep gas flow rate of 2.0 m^3^/h. Fourier transform infrared spectroscopy and gas chromatography-mass spectrometry methods were used to examine the composition of the pyrolysis oil. The pyrolysis oil is rich with aliphatic, aromatic, phenolic, and some acidic chemicals. The physical characteristics of pyrolysis oil showed higher heating value of 19.76 MJ/kg. The char and gaseous components were also analyzed to find its suitability as a fuel.

## Introduction

The lack of energy has turned into a threat to the economic development of countries all over the world due to continuous population growth and industrialization. Biomass is now considered as a resource that may be used indefinitely and has great potential for the generation of power and heat. It can be converted into different forms using thermochemical and biochemical conversion processes^1^. In order to meet current emission limits, it is crucial to reduce emissions of greenhouse gases and harmful pollutants, which can be accomplished by using biomass fuels to produce energy^2^. Use of biomass permits us to improve the part of renewable energy for replacing conventional fuels. The availability of surplus biomass all over the world gives us confidence to utilize it for energy recovery. Almost 30% of waste biomass is only used for energy recovery, while the remainder is disposed of in landfills. Waste biomass typically requires pretreatment to improve its energy characteristics. Commonly, waste biomass materials are used for direct burning due to their lower density and higher moisture level^3^. Combustion, pyrolysis and high-pressure liquefaction are the well-known conversion processes^4^. Among other processes, pyrolysis is the attractive technology used for all kinds of biomass. It is a novel process carried out with no oxygen or air environment^5^. According to the large quantity of literature, it can be known that the pyrolysis process can be conducted 350 to 800°C^6–9^. During pyrolysis, the samples were heated to release organic and inorganic gas elements due to a thermal cracking reaction. Condensable gases and char are the primary products of pyrolysis. The condensable volatiles are further broken to get different gases. The composition of biomass is an important one to determine the value of the pyrolysis products. Pyrolysis oil is a liquid product produced through the condensation of condensable volatiles obtained from pyrolysis. They are renewable liquid products with less sulphur and nitrogen content^10^.

Like forestry and wood-based biomass, agricultural residues are being recognized as a worthy feedstock for pyrolysis^11^. Previously many literatures have considered agricultural residues for pyrolysis oil production. In India, around 650 MT of agri-based residues are generated every year. They are recognized as a potential feedstock that supports the development of the country’s economy^12^. The disposal of agricultural residues from the field is still a major issue since the majority of them are fired in the open atmosphere. In addition to harming the soil and air, it also causes the majority of agricultural waste to be wasted without recovering its chemical components^13^. The burring of 1 kg of agricultural reissues emits approximately 1400 kg of CO_2_ into the environment^14^. These pollutants are the main reason for global warming and other environmental problems^15^. So it is very essential to report the issue associated to the disposal of agricultural residues by finding suitable conversion techniques. Panda et al.^16^ reported a production of 81.2 wt% of pyrolysis oil from kaner seeds at 600 °C. Apart from that, the authors also utilized the seeds obtained from cotton, mahua and mango. Colantoni et al.^17^ used grape vine and sunflower husk for the production of char through pyrolysis under a fixed temperature of 400 °C and 500 °C. The result showed a higher production at 425 °C and it was reduced with increased temperature. The residues from cassava plantations were pyrolyzed by Pattiya and Suttibak^18^ in a nitrogen environment. They looked at the change in yield with respect to various process parameters. According to the findings, at around 475 °C, the residues were pyrolyzed effectively to produce maximum pyrolysis oil. This study produced about 69.1 wt% of oil. In order to assess the yield, Yanik et al.^19^ performed fast pyrolysis on corncob, straw, and oreganum stalks at the temperature of 500 °C. They described that 41 wt% of pyrolysis oil was produced. Madhu et al.^20^ investigated flash pyrolysis of cotton shell residues and produced 51.25 wt% of oil with higher calorific value of 19.32 MJ/kg. The compounds found in the oil can also be used for medicinal purposes. The properties of oil obtained from *Nannochloropsis gaditana* were investigated by Adamczy and Sajdak^21^. According to their findings, a production of 40 wt% pyrolysis oil was recorded at 600 °C, with the majority of olefins.

*Sesbania grandiflora* is a thin tree which is known as the "vegetable hummingbird." This tree is originated to Maritime Southeast Asia and is widespread all over India, Malaysia and Indonesia. Regionally, it is known as katurai and agathi. Sterols and saponins have been identified in the leaves and flowers of this tree^22^. These elements have a good reputation in human health and are found to have favorable biological properties such as antiurolithiatic, anticonvulsant and anxiolytic properties^23^. The leaves of this plant are used as food for humans and animals. They are also used in Ayurvedic medicine to alleviate epileptic seizures. The raw juice is used to treat fever, itching and worms. The powdered roots are used to cure swelling. This tree can reach up to 2 m tall within 100 days. It can yield up to 27 kg of green leaves every 3 to 4 months. The wood bark of this tree is around 20 cm in diameter and it is used for burning purposes. The plants cultivated in the rural sector are destroyed in the field itself by open fire. The burning of this kind of plant in the field is destroying the atmosphere with its numerous gas emissions. Researchers from many academic institutions in India have been looking at the possibility of pyrolyzing agricultural residues to produce biofuels and chemicals. They are playing a significant role in the development of the bioenergy sector. Lab-scale reactors for agricultural residues have been developed by many scientific people. The pyrolysis process for converting agricultural residue has adequate operational experience through lab-scale investigations for determining its economic viability.

This work deals with the pyrolysis of *S. grandiflora* residues for pyrolysis oil production. The yield and characterization at different operating parameters were the focus of this investigation. The experiments were conducted at various temperatures, particle sizes and sweep gas flow rates of 350–550 °C, 0.5 mm to 2.0 mm and 1.5–2.25 m^3^/hr respectively. The selected parameters were selected from past literature and are well suitable for the conversion of agricultural residues to yield maximum pyrolysis oil^24,25^. This work is considered unique in terms of the selection of feedstock. This study ended with the analysis of pyrolysis oil, gas and char particles. Different analytical methods were employed to characterize the pyrolysis oil, and the physicochemical parameters were assessed according to American Society for Testing and Materials (ASTM) standards.

## Materials and methods

### Materials

The feedstock used for this analysis is agricultural residue obtained from a mature tree of *S. grandiflora*. The waste *S*. *grandiflora* residues that gathered from Saveetha farm house, Chennai, Tamil Nadu, India, were utilized in this research. It is said that no samples taken from the wild or from the agricultural lands by considering the national or worldwide concern. Every procedure was carried out in compliance with the applicable national and international norms, legislation, and recommendations. As a naturally occurring plant, *S. grandiflora* is widely used in the production of charcoal. It is a fast-growing tree. The flowers and leaves are used for cooking and eaten as vegetables in Southeast Asia and South Asia. Among all the green leafy vegetables, the leaves are the best source of vitamin A, calcium, and phosphorus. This leaf is the ideal dietary supplement to support bone health and immunity. Due to these medicinal and regular uses, the plants are cultivated throughout southern India. The wood of the mature trees is burned in the field itself and sometimes utilized for the production of charcoal. The open burning of the residues poses a serious threat to the environment. The wood burns with a lot of smoke, making it a low-quality fuel. It is also rather light. It burns quickly but produces little heat because its weight per square meter is only 500 kg/sq. It is a locally popular fuel wood, even though the wood needs to be well dried because it degrades in storage and turns dusty, corky and unsuitable for burning^26^. Concerning environmental issues, the study selected *S. grandiflora* as the feed material for the flash pyrolysis process.

The tree is adapted to hot and humid environments. It may be cultivated on a variety of soil types, including those with poor drainage and waterlogging. The tree is native to Asia, specifically India, Malaysia and the Philippines. At present, the tree grows many parts of the world including Mexico, West Africa, Cuba, Kenya, USA, Uganda, Nigeria, South Africa, South America and Nepal. The plant is widely grown in India, particularly in the states of Tamil Nadu, Andhra Pradesh, Kerala, Assam, Gujarat, and West Bengal. They may spread like weeds in small spaces and flourish in hot, humid climates^26^. From the tree, roots, leaves and flowers were removed and cleaned. The solid wood material was cut into smaller particles using a wood cutter. Initially, the larger wood particles were cut into smaller particles using a manual wood cutter. After that, the residues were ground in a ball mill (Emkad Enterprises, Coimbatore, India) and sieved to get uniform particle sizes of 0.5, 1.0, 1.5 and 2.0 mm. For ball milling, the weight of the balls to the sample was maintained at 20:1, and the ball milling period was 1 h..

### Material and product characterization

The residues were characterized through proximate and ultimate testing. The thermogravimetric analysis (TGA) on the residue was done using TGA701 analyzer. The TGA analysis was done at 800 °C (heating rate at 15 °C/min) under nitrogen environment. The physical characteristics of the pyrolysis oil were conducted using a standard viscometer, pH meter, flash point apparatus and a calorimeter. The FT-IR study of the pyrolysis oil was done by BRUKER TENSOR 27 FTIR spectrometer at a range of 4000 and 400 cm^-1^. The GC–MS study of the oil and gas was found by Thermo GC—TRACE Ultra Ver: 5.0, Thermo MS DSQ II. For this analysis, the column (DB-35) of dimensions of 30 m × 0.25 mm × 0.25 µm was set initially at 60 °C. It was held for 2 min and raised to 250 °C at a rate of 10 °C/min. Helium with 99.9995% purity was employed for the analysis. The mass spectrometer (MS) was operated with an ion source temperature of 200 °C in the range 40–600 m/z. The National Institute of Standards and Technology (NIST) library identifies the chemical elements according to the peak obtained from GC. The component analysis of the char was also identified using the same carbon, hydrogen, nitrogen and sulphur analyzer.

### Reactor facility and procedure

All the experiments were conducted with a material feed rate of 25 g/min. The system is made up of a cylindrical chamber of diameter 50 mm with total length 1 m and surrounded by an electrical heater. Inside the reactor, the fluidization was created by using nitrogen gas. For better fluidization and heat transfer, a required quantity of sand is used along with nitrogen. The flowrate of the gas was controlled by a valve attached to the rotameter. The bed temperature was checked with five K-type thermocouples. The bed is heated electrically and insulated properly with Chromel Alumel and wool. The cyclone separator fitted in the setup is used to separate dust and char particles. The experimental work was carried out in three different phases. Initially, the analysis was done by changing temperature. The point at which the maximum pyrolysis oil yield was produced was noted for further experimental work. For second and third phases, the size of biomass material and sweeping gas flow rate were varied from 0.5 to 2.0 mm and 1.5 to 2.25 m^3^/h, respectively. At the end of the experimental work, the optimized parameters to yield maximum pyrolysis oil were noted. The pyrolysis oil, gas and char produced at that point were considered for characterization analysis. The mass of oil and char obtained at a single run was found by a weighing the product and the mass of gas were measured using remaining material balance.

## Results and discussion

### Biomass characterization

Before the experimental work, the samples were dried in a closed furnace maintained at 100 °C for 1 h and sieved to get different diameter. The initial characterization were done on the feedstock and described in Table 1. From the table, it is evident that the residues have higher volatile matters. Almost 70 wt% of the materials can be volatilized during pyrolysis. The moisture content of the material is 7.22 wt%. It can be further reduced by drying. The moisture in the feedstock affects the pyrolysis experiment and yield quality. The ash in the residue is a minimum of 6.61 wt%. According to Gray et al.^27^, the lower ash is advised for the pyrolysis, since the higher ash percentage strongly affects the pyrolysis oil quality. It was discovered that ash concentrations of up to 3 wt% in pine wood were sufficient to alter the biomass pyrolysis distribution and its composition. The yields of the pyrolysis were decreased if the ash percentage is higher^28^. According to the observations, it is suggested to choose biomass with an ash level of no more than 10 wt%.

**Table 1:** Characteristics of *Sesbania grandiflora* residues (%).

Parameters	Value
Proximate analysis (wt%)	
Volatile matter	68.78
Fixed Carbon^a^	16.33
Moisture Content	7.22
Ash	6.61
Ultimate analysis (wt%)	
C	43.70
H	6.03
N	3.80
O^a^	46.44
S	0.03
Lignocellulosic content (%)	
Cellulose	61.75
Hemicellulose	18.70
Lignin	17.55

^a^By difference.

### TGA analysis

**Figure 1:**
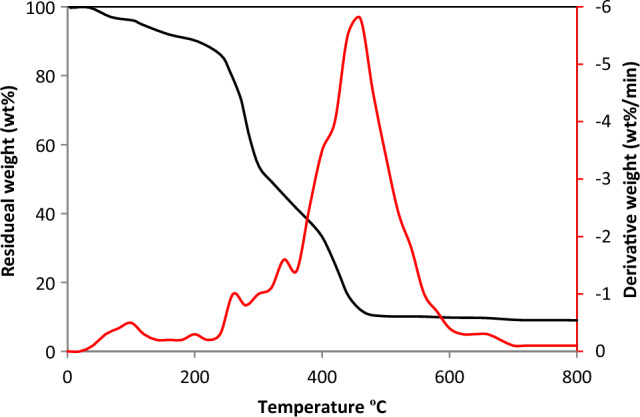
TGA and DTG analysis of *Sesbania grandiflora* residue.

After physical evaluation of the residues, the TGA analysis of the material was done to evaluate their thermal behaviour. Typically, cellulose, hemicellulose, lignin and some inorganic elements present in the biomass have some unique thermal behaviour. Yang et al.^29^ presented that the decomposition of these elements ensued at the temperature ranges between 200 and 315 °C, 315 °C to 400 °C, and 160 °C to 900 °C, respectively. In TGA study, the primary weight loss is linked to the evaporation of water particles. In general, the primary pyrolysis process for decomposing lignocellulosic biomass occurs between 225 and 440 °C, where cellulose and hemicellulose were broken down. This zone is attributed to devolatilization of biomass particles. The hemicelluloses are the most heat-sensitive materials. The decomposition of hemicellulose plays a vital role during pyrolysis due to the existence of xylans, which are the most reactive^30^. Cellulose in the residues is devolatilized in two phases. The initial phase is endothermic occurred from 225 to 340 °C to form char, tars and carbon oxides. The second phase of cellulose decomposition is related to exothermic depolymerization, which occurs after 340 °C. The decomposition of biomass residues occurred in two stages: in stage 1, breakdown of cellulose occurs and in stage 2, breakdown of hemicellulose occurs. The breakdown of these two is the key process for producing volatile products. The decomposition of hemicelluloses yields more gas products than other elements. Xylose in the hemicellulose is the cause for the production of acids and non-condensable gases during pyrolysis^31,32^. According to Hubble and Goldfarb, xylose and cellulose combine homogeneously and release the gases rapidly from the matrix within a short duration for heterogeneous reactions^32^. Lignin begins to degrade at 175 °C and continues to degrade up to 800 °C. This process is best known for producing ash content in oxidizing environments^33^. In this study, the decomposition of the residue started around 40 °C and proceeded until 140 °C with maximum weight loss of 10.5%, the initial weight loss representing the evaporation of water molecules and hydrolysis of extractives. Following this, the maximum weight loss of 27.5% achieved between 240 and 375 °C. The final decomposition occurred between 375 and 470 °C. In the middle stage, cellulose and hemicellulose started to break down, followed by lignin. The degradation reaches its maximum level at 462 °C, with a 65% weight loss. The largest weight loss was primarily caused by the degradation of cellulose and hemicelluloses. These results indicated that the majority of weight loss occurred below 600 °C due to hydrocarbon volatilization^34^. Consequently, for further experimental work, the reaction temperature was set from 350 to 550 °C. The TGA and derivative thermogravimetry (DTG) pattern of *S. grandiflora* residue is given in Fig. 1.

### Yield analysis

#### Effect of temperature

**Figure 2:**
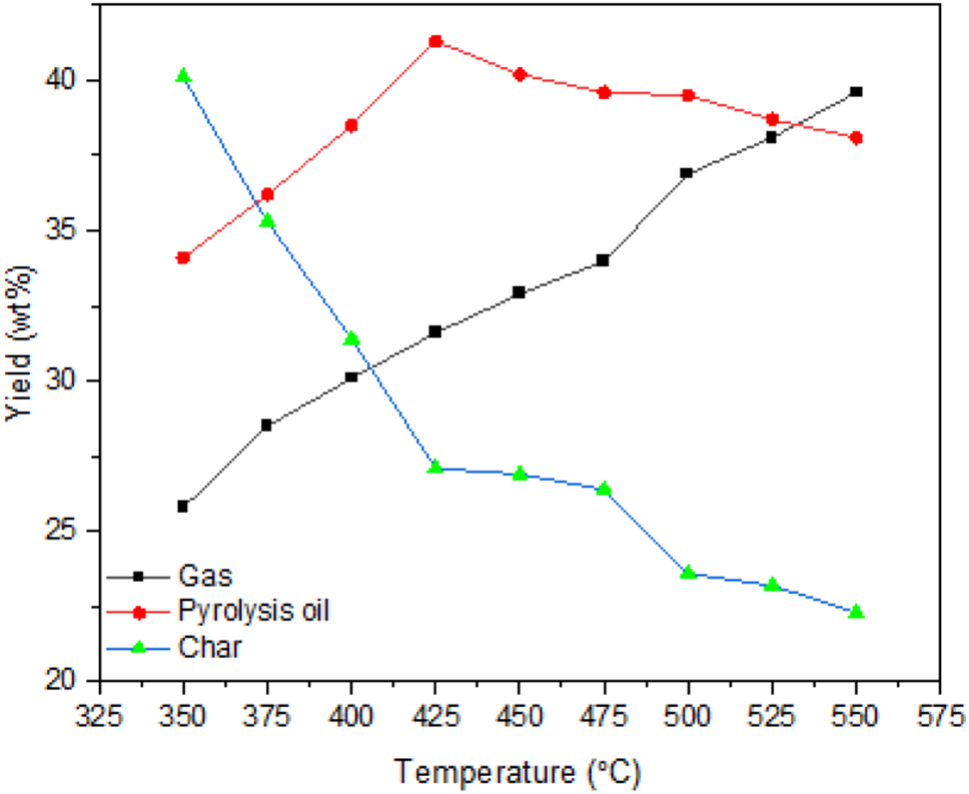
Effect of temperature.

Figure 2 shows product yields obtained between 350 and 550 °C. In this phase, other two parameters were kept at 0.75 mm and 1.5 m^3^/h respectively. The temperature of the bed significantly affects the yields of the reaction. The conversion of feedstock to the valuable biofuel has increased with increased temperature. As seen in the figure, the char production was declined from 41.1 to 22.3 wt% when changing the bed temperature from 350 to 550 °C. Over the entire process, the higher char yield was observed 350 °C. Compared to the initial char yield, the yield of char was reduced to 45.7% at an elevated temperature. At higher temperature, the breakdown of the residues or secondary decomposition of the char improves the production of condensable volatiles^35^. The same trend was observed in the literature^36,37^. The yield of gas is improved with increased reactor temperature, just the opposite to the char yield. At 350 °C, the gas yield was 26.8 wt%. At 550 °C, the gas yield was increased to 39.6 wt%. The total gas yield was increased by up to 47.7% during this phase. The secondary decomposition of pyrolysis vapours and char element at elevated temperature is the causes for increased gaseous products^38^. Based on the findings of Yang et al.^39^, cellulose and hemicellulose release more condensable volatiles than lignin. The high mineral content of the residues may also have an impact on the overall volume of product gas. According to Couhert^40^, minerals present in the biomass reduce the activation energy, which speeds up the exothermic reactions. As reported earlier, the presence of ash in the feedstock adversely affects the pyrolysis experiment. The samples with higher lignin content yield more char during pyrolysis because, up to 500 °C, cellulose decomposes fully and only 20% of the hemicellulose decomposes, whereas lignin decomposes only to about 60%^41^. For pyrolysis oil yield, the yield of oil increased gradually to 41.3 wt% and then decreased to 38.1 wt%. This trend is recorded till the bed temperature was increased to 425 °C after that the yield was decreased. The higher reaction temperature enhanced the conversion of carbon into gas^42^. The maximum production of pyrolysis oil was recorded at 425 °C. Between 400 and 500 °C, the maximum conversion of biomass to pyrolysis oil was obtained due to the release of condensable volatiles. From the decrement of oil products at higher temperatures, it can be understood that the secondary reaction of the liquid volatiles and the continuous decomposition of char products preceded the yield of non-condensable volatiles^43^.

#### Effect of particle size

**Figure 3:**
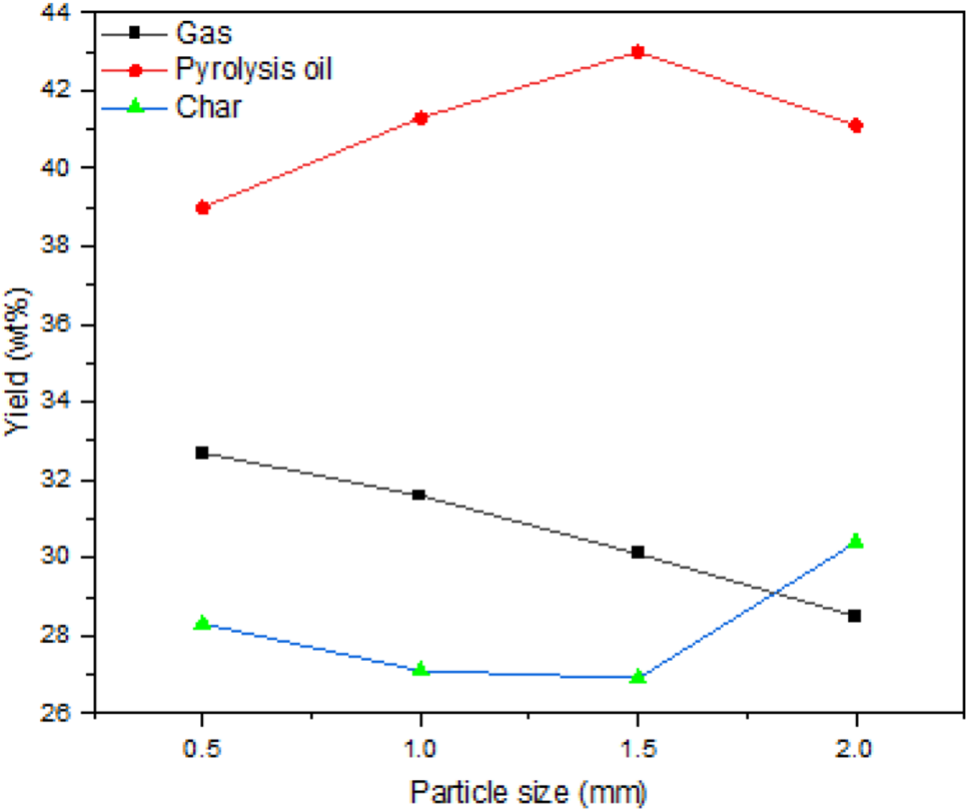
Effect of particle size.

Figure 3 depicts the production of pyrolysis oil, char and gas obtained at 425 °C with respect to different particle sizes. In this experimental section, with a char production of 26.9 wt% under 1.5 mm size, a maximum of pyrolysis oil was obtained. The pyrolysis oil yield was 39.0 wt% with a char yield of 28.3 was observed at the lowest particle size of 0.5 mm. The weight percentage of pyrolysis oil, char and gas at larger particle size was 41.1, 30.4 and 28.5 respectively. In the pyrolysis of rapeseed and tea waste, the particle size significantly affected the product characterization^44,45^. In some cases, the influence of particle size on pyrolysis process is moderate^46^. When the particle is admitted into the reactor, at the initial stage, the temperature profile nearer to the wall of the particle is quite steep, but as time progresses, it becomes less steep. This can be elucidated by the fact that the earliest phases of pyrolysis show higher heat transfer resistance near the wall when heat is transferred by convection and radiation from the wall surface. On the other hand, when the heat is transferred only in convention mode, the heat transfer along the wall is lower^47^. Heat is transferred inside the biomass particles through a combination of conduction, radiation and transmission through the gas phase. For simplicity, only the conduction mode of heat transfer is considered for the analysis. Under control volume, during heating, the following equation represents the energy balance^48^:

1 $$\frac{{D\left( {\rho C_{p} T} \right)}}{{D_{t} }} = - \left( {\nabla \cdot q} \right) + \left( { - \Delta H} \right)\left( {\frac{{ - \partial_{\rho } }}{{\partial_{t} }}} \right)$$

If the heat is transfereed in one dimensional, the Fourier law is described as

2 $$q_{r} = - k\left( {\frac{\partial T}{{\partial r}}} \right)$$

Equation (1) becomes

3 $$\frac{{\partial \left( {\rho C_{p} T} \right)}}{{\partial_{t} }} = \frac{{\left( {\frac{1}{{r^{p} }}} \right)\partial \left[ {r^{p} k\left( {\frac{\partial T}{{\partial r}}} \right)} \right]}}{\partial r} + \left( { - \Delta H} \right)\left( {\frac{{ - \partial_{e} }}{{\partial_{t} }}} \right)$$

Under control volume if density (ρ), Specific heat (Cp) and thermal conductivity of the feedstock are assumed to remain constant, Eq. (3) becomes:

4 $$\rho C_{p} \left( {\frac{\partial T}{{\partial t}}} \right) = k\left[ {\left( \frac{p}{r} \right)\left( {\frac{\partial T}{{\partial r^{2} }}} \right)} \right] + \left( { - \Delta H} \right)\left( {\frac{{ - \partial_{\rho } }}{{\partial_{t} }}} \right)$$

A symmetric particle in a cylinder shaped furnace with nitrogen gas has the initial and boundary conditions shown below:

5 $$t = 0, T\left( {r, 0} \right) = T_{0}$$

6 $$t > 0, r = 0, \left. {\left( {\frac{\partial T}{{\partial r}}} \right)} \right|_{r = 0} = 0$$

7 $$r = R, - k\left. {\left( {\frac{\partial T}{{\partial r}}} \right)} \right|_{r = R} = h\left( {t - t_{f} } \right) + \in \sigma \left( {T^{4} - T_{f}^{4} } \right)$$

In the above equation, external heat transfer is considered to be the combination of convection and radiation. The feedstock size is the key parameter for heat transfer enhancement during pyrolysis. The material with a smaller particle size can be heated more quickly than a large particle. If the diameter of the feedstock is greater, the time taken to transmit heat from outer layer to the inner core will be at its maximum, which leads to the formation of char^49^. At moderate particle size, the material can be heated uniformly, leading to maximum pyrolysis oil output. Pyrolysis of *S. grandiflora* at 1.5 mm size seems to be suitable for maximum oil yield. The particle size was statistically significant in this work to yield maximum pyrolysis oil. At 425 ºC, when the size of the particle was maintained at 1.0 mm the yield of pyrolysis oil was 41.3 wt%. On the other side, when the particle size was changed to 1.5 mm the yield of pyrolysis oil was increased to 43.0 wt%, which is 4.12% more. While considering gas yield, changing the particle size from 1.0 to 1.5 mm reduced the yield by 4.75%. At 0.5 mm size, the production of pyrolysis oil was 39.0 wt% with maximum gas production of 32.7 wt%.

#### Effect of sweep gas flow rate

**Figure 4:**
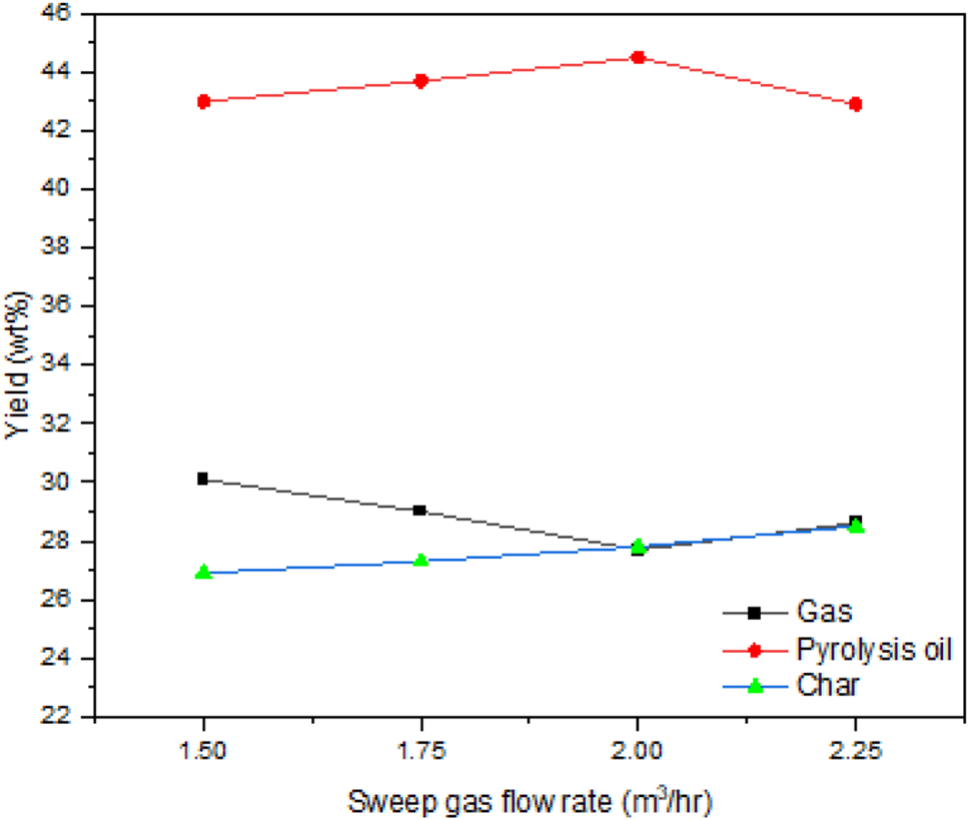
Effect of sweep gas flow rate.

In this part, the sweeping gas flow rate was changed as 1.5, 1.75, 2.0 and 2.25 m^3^/h with fixed bed temperature (425 °C) and particle size (1.5 mm). The experimental results are displayed in Fig. 4. During fluidized bed pyrolysis, the residence time of the vapor phase is influenced by the flow of sweeping gas. The proper gas flow rate should be used because an improper gas flow rate during the experiment impacts the residence time and minimizes the secondary breakdown reaction. The higher sweep gas flow affects the residence time of the evolved gases and eliminates the products from the bed quickly, which reduces the secondary decomposition processes^50^. In this work, the flow of nitrogen didn’t affect the product yield significantly. The findings of Westerhof et al.^51^ and Kim et al.^52^ are also consistent with this. By changing sweeping flow rate from 1.50 to 2.0 m^3^/h, the oil yield was changed from 43.0 to 44.5 wt%. The reduced oil beyond 2.0 m^3^/h may be the reduction of contact time, area and partial pressure of each condensable component within the reactor. According to Uzun et al.^53^, the maximum pyrolysis oils are expected when the developed pyrolysis vapors are immediately removed with higher flow rate with reduced vapour residence time.

### Characterization of pyrolysis oil

#### Physical analysis

The reactor condition and method of pyrolysis under which pyrolysis oils are produced have a strong effect on the structure of those oils. The properties can be varied according to the feedstock used and its composition^54^. The physical and elemental composition of the produced pyrolysis oil at its maximum yield point is displayed in Table 2. The level of oxygen in the pyrolysis oil is found to be lower than that in the feedstock. The higher oxygen content of the oil indicates that it has been exposed to the atmosphere for a longer period of time. The presence of the oxygenated elements causes the oil to be acidic, causing subsequent harm such as corrosion. In addition to that, liquid fuel with higher oxygen content can’t be used as a fuel for transportation since it reduces the efficiency of the engine. Anyhow, the presence of oxygen can be further reduced by various chemical processing methods such as hydrotreatment, catalytic cracking, emulsification, and steam reforming^55–57^. These techniques convert the oxygenated components into light hydrocarbons, aromatics, hydrogen and syngas. Hydrodeoxygenation, ketonization, condensation, aromatization and cracking are some of the chemical reactions involved in the upgrading process. For instance, in pyrolytic cracking, hydrogen exchange, aromatic side-chain scission, isomerization and deoxygenation are the key processes involved in the pyrolysis oil. The higher oxygen molecules indicate the presence of high polar groups, which leads to a viscous fluid with a higher boiling point. The pyrolysis oil produced in this study shares characteristics with pyrolysis oil made from other feedstocks^58,59^. Compared to pyrolysis oil derived from hard wood and *Scenedesmus dimorphus*, pyrolysis oil derived from *S. grandiflora* has higher calorific value. The lower viscosity of the derived pyrolysis oil compared to tea waste and hard wood pyrolysis oil leads to better atomization properties. The pH value of the pyrolysis oil derived in this study was lower. Low-pH pyrolysis oils are reportedly extremely corrosive to materials made of aluminum, mild steel and nickel. Pyrolysis oils as a renewable fuel still have limited commercial application, mainly due to their lower heating value than fossil diesel and the difficulty of mixing the raw oils with the present petroleum-based transportation fuels^60^. However, the valuable compounds present in the pyrolysis oils have been extensively utilized for various chemical industries^61^. The quality of the pyrolysis oil is believed to be positive. The pyrolysis oil can maintain its homogeneity for at least six months when it is stored at atmospheric temperature^62^. However, before pyrolysis oil can be commercialized, its overall production costs must be reduced, its quality must be increased and its ability to produce homogeneous liquid must be proven on an industrial scale. In order to improve the quality of the pyrolysis oil, additional experimental analysis has to be done. Alcohol addition and solvent addition have been suggested to improve its quality^62^.

**Table 2:** Properties of the pyrolysis oil.

Component	Value [this study]	Tea wastes^45^	Hard wood^63^	*Scenedesmus dimorphus*^64^	Palm shell^65^	Diesel^66^	Unit
Density	975	980	1220	841	1051	850	kg/m^3^
Viscosity	3.9	4.1	13	$	3.2	3.9	cSt
Flash point	145	135	66	40	$	57	°C
pH	4.3	4.8	$	5	2.5	–	–
C	50.35	51.33	55.5	74.73	19.48	86.5	wt%
H	6.98	7.63	6.7	10.6	8.92	13.2	wt%
N	5.04	5.14	0.1	5.79	0.2	0.02	wt%
S	0.03	0.02	0.0	0.61	0.04	0.24	wt%
O^a^	37.60	35.88	37.7	8.27	71.40	–	wt%
Heating value	19.76	21.34	17.5	28.52	6.58	43.60	MJ/kg

^$^Not reported.

^a^By difference.

#### FT-IR analysis

**Figure 5:**
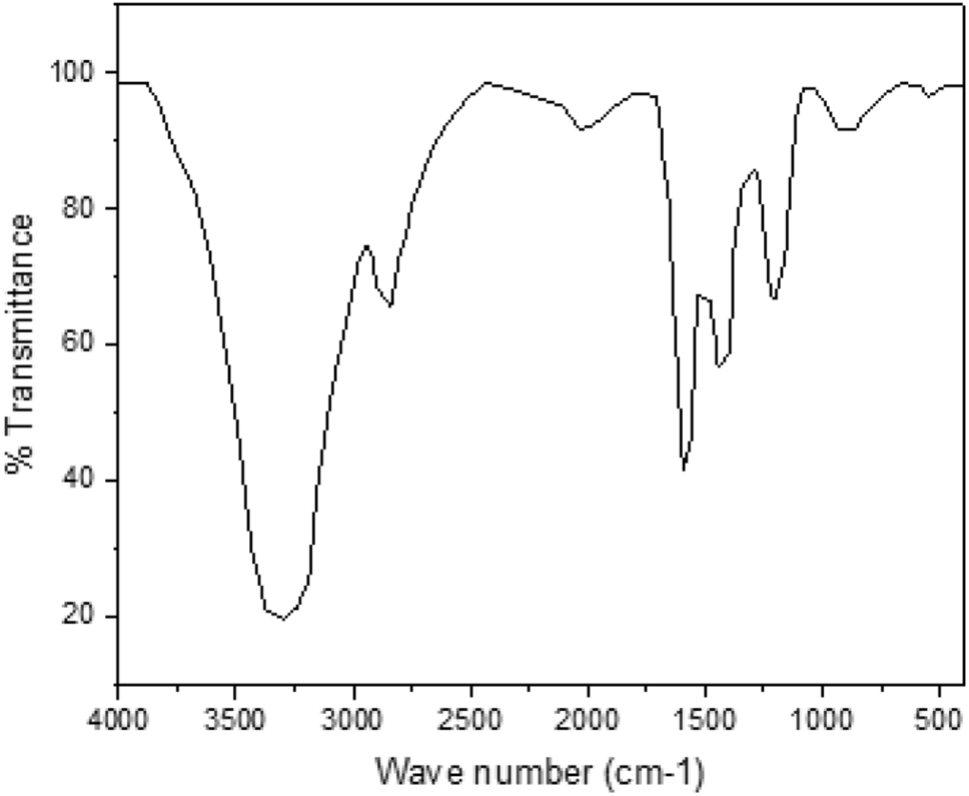
FT-IR analysis of pyrolysis oil.

FTIR spectroscopy has become a beneficial method for determining the potential engagement of biomolecules. The spectrum of the obtained pyrolysis oil is revealed in Fig. 5. The investigation was conducted on KBr pellet. The existence of a strong intense band at 3372.5 cm^−1^ exhibited the occurrence of O–H group components, which indicate the existence of alcohols, phenols and oxygenated components in the pyrolysis oil^67^. The presence of oxygenated components is common in biomass derived pyrolysis oil due to the higher oxygen content of the feedstocks. The alkane group component is identified by the absorbance peak of C–H intense at 2901.6 cm^−1^. The same components also appear at the absorbance peak between 1400 and 1300 cm^−1^ in the pyrolysis oil derived from municipal solid wastes^68^. The absorbance of stretching vibrations of C=C peaks at 1589 cm^-1^ may be a sign of alkenes in the pyrolysis oil. The presence of aromatics in the oil is also indicated by this absorbance peak. The same peak was also observed in the pyrolysis oil derived from Pine Wood^69^. The C–H bend at 1454.7 cm^-1^ also confirmed the existence of methyl and methylene groups of alkanes in the oil. The O=C stretch and = C–H bend at 1219.3 cm^−1^ and 931.7 cm^−1^ showed aliphatic and alkenes in the pyrolysis oil.

#### GC–MS analysis

The various compounds in the unfractionated pyrolysis oil were found in this analysis. Table 3 represents the total chemical components identified in this analysis. The majority of the substances were phenolic and aromatic. It is also confirmed with FTIR analysis. The existence of acids in the pyrolysis oil creates adverse effects during storage hence it is undesirable. Phenol is the major element identified in the pyrolysis oil with the highest peaks. Phenol (C_6_H_6_O), 2-methoxyphenol (C_7_H_8_O_2_), 3,5-dimethyl phenol (C_8_H_10_O) and 1,6-anhydro-beta-D-glucopyranose (C_6_H_10_O) are the chief elements identified in this analysis. Other than these elements, oil is composed of many oxygenated chemicals. The breakdown of oxygenated compounds in the feedstock is the cause for the existence of these compounds. These outcomes are in line with Ingram et al.^70^ and Rao et al.^71^.

**Table 3:** GC–MS analysis of the pyrolysis oil.

Name of the compound	RT (Min)	Molecular formula	Molecular weight	Area %
3-Methyl-1-phenyl-1H-inden	3.76	C_16_H_14_	206	1.04
2-furanmethanol	4.11	C_5_H_6_O_2_	98	2.52
3,4-dimethylbenzoic acid	5.72	C_9_H_10_O_2_	150	3.08
2-methoxy-4-propylphenol	5.90	C_10_H_14_O_2_	166	4.44
2-Methyl- Furan	6.78	C_5_H_6_O	82	1.82
Naphthalene	9.40	C_10_H_8_	128	0.73
Phenol	9.94	C_6_H_6_O	94	15.54
Oleic acid	10.13	C_18_H_34_O_2_	282	3.47
Methyl-pyridone	11.05	C_6_H_7_N	109	2.55
2-Acetyl furan	12.08	C_6_H_6_O_2_	110	2.11
3-Undecene, (Z)-	13.58	C_11_H_22_	154	0.86
2-methoxy-4-(1-propenyl)phenol	15.54	C_10_H_12_O	164	3.50
1,6-anhydro-beta-D-glucopyranose	15.71	C_6_H_10_O	162	4.01
1,3-Pentadiene, 2,4- dimethyl	16.27	C_7_H_12_	96	2.57
Hexancdioic acid, bis(2-methylpropyl) ester	17.01	C_14_H_26_O_4_	258	3.40
Linalyl 2-Methylpropanoate	18.55	C_14_H_24_O_2_	224	2.01
Acetic acid	19.76	C_2_H_4_O_2_	60	1.58
Ethisterone	20.20	C_21_H_28_O_2_	312	2.97
2-Phenyl-1-p-tolylethanol	20.87	C_14_H_16_O	210	0.88
Phenol, 3,5-dimethyl-	22.33	C_8_H_10_O	122	6.44
1,2-Bis(2'-quinolylmethyl)ethylene	24.60	C_20_H_14_N_2_	282	2.50
3-Hydroxy-4-methoxybenzoic acid	25.03	C_8_H_8_O_4_	168	2.00
Pyridine 2-methyl	26.40	C_6_H_7_N	93	3.40
2-methoxyphenol	27.88	C_7_H_8_O_2_	124	7.49
Azulene	28.63	C_9_H_8_	128	0.83
Tetradecane	29.74	C_14_H_30_	198	2.96
Phenol, 4-amino-	30.41	C_6_H_7_NO	109	3.31
4-Ethyl-2-methoxy phenol	30.82	C_9_H_12_O_2_	152	3.46
1-Methyl-1,3,3-triphenylindan-2-one	31.47	C_28_H_22_O	374	2.01
2-Furancarboxaldehyde,-5-Methyl	33.39	C_6_H_6_O_2_	110	2.57
1-Ethynyl-1,2,3,4-tetrahydro-á-carboline	34.25	C_13_H_12_N_2_	196	0.80
Hexadecanoic acid, methyl ester	36.07	C_17_H_34_O_2_	270	1.56

### Analysis of char

The char obtained in this study was analyzed by proximate and elemental analysis and presented in Table 4. The volatile matter is identified as 22.82 wt%. Antal and Grnli^72^ claimed that the presence of volatiles in the char is a carbonization measurement that is related to the process temperature. The amount of volatile matter can typically range from 5 to 40%. The volatile matter found in the char (22.82%) has been recorded between the recommended values. The value of fixed carbon is 67.31 wt%, which is also between the suggested values of 65 to 90%. The elements identified in the char were compared with biomass, and it was noticed that the carbon in the char is higher than the feedstock. Additionally, it was noted that H, N, S and O contents, differed significantly compared to feedstock. This is due to the expulsion of O and volatile matter from the feedstock, which improved the quantity of C in char^73^. The analysis showed higher calorific value of the char than the feedstock material.

**Table 4:** Analysis of the char in %

Parameters	*Sesbania grandiflora*	Wood saw dust^74^	Grape bagasse^75^	*Albizia odoratissima*^76^
Volatile matter	22.82	21.57	$	24.52
Fixed Carbon	67.31	65.63	$	68.31
Moisture Content	3.11	1.12	$	2.12
Ash	6.94	11.86	$	5.05
C	63.76	63.48	72.09	52.41
H	3.28	2.86	3.05	7.54
N	3.94	0.72	1.4	3.22
O^a^	28.63	32.94	23.46	36.62
S	0.39	$	$	0.21
Empirical formula	CH_1.783_N_0.067_O_0.429_	CH_0.54_N_0.01_O_0.38_	CH_0.51_N_0.02_O_0.24_	$
Heating value (MJ/kg)	21.72	22.03	28.40	23.47

^$^Not reported.

^a^By difference.

### Analysis of gas

The pyrolysis gas obtained from *S. grandiflora* at 425 °C has the following compositions: 3.46% CO, 7.43% CO_2_, 8.37% H_2_, 1.09% O_2_ and 3.75% CH_4_. The existence of H_2_ and CO in the pyrolytic gas can be used to produce liquid fuels. The formation of CO in the gas fraction is due to the reverse Boudouard reaction^77^. The heating value of raw gas fractions was assessed as 9.45 MJ/kg. It can be improved by eliminating CO_2_ content. In general, it may be noted that the pyrolysis gas contains up to a maximum of 15% methane and other hydrocarbons. The pyrolysis gas produced from *S. grandiflora* contains 3.75% methane. It is a combustible gas that can be used for the production of electricity and other organic chemicals. Additionally, hydrogen is one of the most common pyrolysis gas constituents, and its volume percentage varies from a few to a few dozen. Pyrolysis of waste tyres and plastics can yield more hydrogen components than biomass pyrolysis. Temperatures between 400 and 700 °C were used by Berruecco et al.^78^ to generate hydrogen concentrations between 2.6 and 17.8 vol% at 550 °C. Lopez et al.^79^ achieved 22.27 vol% of hydrogen in pyrolysis gas. The residual gases produced in this study can be used for electricity production. In order to compare the technologies taken into consideration in this work, it is also possible to do a preliminary economic analysis of the usage of the pyrolysis gas. Leme et al.^80^ evaluated the usage of pyrolysis gas produced in charcoal manufacture for electricity generation. The study showed a total electricity production of 0.93 MWh per tonne of charcoal produced through the use of pyrolysis gas.

### Cost analysis

Cash flow and capital cost analysis are the two basic pillars of economic analysis. This research will be used to calculate the cost of producing pyrolysis oil as well as the annual investment needed to run a production plant^81^. The price can be determined by capacity factoring (heat and mass balances, power supply, size), equipment-based assessment and vendor quotation. There are a number of barriers that must be addressed to get an attractive and profitable return from the pyrolysis oil plant. In relation to that, improving product yield as well as reducing the quantity of heat loss during the process should be taken into account as essential variables for the pyrolysis oil production setup. To increase the value of pyrolysis oil, an upgrading technique should be developed. For long-term access to this resource, collaborative efforts with the government sector should continue with funding and support. In addition, it is important to study the environmental problems associated with pyrolysis oil usage in burners, power plants and vehicles for long-term development^82–87^.

## Conclusion

Pyrolysis is one of the possible ways to convert biomass into higher-value products. In this work, *S. grandiflora* residues were pyrolyzed in a fluidized bed reactor to maximize the pyrolysis oil yield. This study is novel in terms of biomass material selection. Here, a maximum of 44.7 wt% of pyrolysis oil was obtained under optimal parameters. Temperature was found to be a significant factor in determining pyrolysis product yield among the selected factors. When the temperature was raised, the oil output initially increased, reaching a maximum at 425 °C. The yield of pyrolysis products was moderately influenced by the particle size and there was no substantial change with respect to sweep gas flow rate. Different characterization studies on pyrolysis oil indicate that it can be utilized as an energy fuel after refining and upgrading. Around 35 major chemical components were identified through chromatography analysis. These chemical components can be used as feedstock for the chemical and pharmaceutical industries. In addition to that, the char can be used as a fuel and as a precursor to activated carbon. This method can contribute to a biorefinery model and increase the range of products that can be produced from biomass. The market size of pyrolysis oil in 2031 is predicted to be USD 481 million. The results of the current study indicate that *S. grandiflora* residue is a promising feedstock for pyrolysis. This statement can be supported if the produced products are utilized effectively.

## Data Availability

The datasets generated during and/or analyzed during the current study are available from the corresponding author and can be shared on reasonable request.
